# Noncommunicable diseases: a call for papers

**DOI:** 10.2471/BLT.18.208843

**Published:** 2018-03-01

**Authors:** Viroj Tangcharoensathien, Titiporn Tuangratananon, Prin Vathesatogkit, Rapeepong Suphanchaimat, Churnrurtai Kanchanachitra, Bente Mikkelsen

**Affiliations:** a International Health Policy Program, Ministry of Public Health, Nonthaburi 11000, Thailand.; b Faculty of Medicine Ramathibodi Hospital, Mahidol University, Bangkok, Thailand.; c Institute of Population and Social Research, Mahidol University, Nakhon Pathom, Thailand.; d Global Coordination Mechanism Secretariat for Noncommunicable Diseases, World Health Organization, Geneva, Switzerland.

The global burden of noncommunicable diseases is high, with 40 million deaths per year, including 15 million deaths in individuals younger than 70 years.^1^ In low- and middle-income countries, noncommunicable diseases account for 78% (31 million) of all deaths, yet only 1% of global health funding goes towards preventing these diseases.^2^ The cost of not taking action against such diseases is enormous, not only in diagnosis and treatment costs, but also in premature deaths. Estimations suggest that economic loss from premature deaths due to noncommunicable diseases will be over 30 trillion United States dollars in the next 20 years, equivalent to half the global gross domestic product in 2010.^3^

To reduce the prevalence of noncommunicable diseases, governments need to implement policies addressing different types of risk factors, including socioeconomic, modifiable behavioural and clinical factors. Population-wide policies and interventions are more effective than clinical approaches at an individual level, which are also more expensive.

Policies addressing modifiable risk factors such as tobacco use, alcohol consumption, unhealthy diet and physical inactivity require effective multisectoral actions for health through a whole-of-government approach^4^ that focuses on population-wide primary prevention.^5^ This approach lowers risks and provides an enabling environment for the adoption of healthier behaviours. For example, ratifying and effectively implementing the Framework Convention on Tobacco Control is a measure to reduce exposure to tobacco. However, governments often find it difficult to safeguard the health of their population. For instance, food and alcohol marketing interferes with public policy on health, especially when exporting countries’ governments promote alcohol and unhealthy food in importing countries.^6^^,^^7^

Noncommunicable diseases are now on the global agenda and have garnered strong political commitments, such as during the United Nations General Assembly high-level meeting on the prevention and control of noncommunicable diseases in September 2011. The Assembly endorsed a declaration urging member states to integrate noncommunicable disease prevention and control into their national agenda. The World Health Organization (WHO) *Global action plan for the prevention and control of NCDs* (2013–2020) was adopted to reduce premature deaths due to noncommunicable diseases by 25% by 2025. Sustainable development goal 3.4 reaffirms the commitment to reduce premature mortality due to noncommunicable diseases by one third by 2030.

By 2017, 94 out of 194 WHO Member States had developed their national action plan on noncommunicable diseases.^8^ However, despite these political endorsements, there is a large policy-implementation gap in translating political commitments into concrete programmatic actions that yield positive outcomes.

The *Bulletin of the World Health Organization* will publish a theme issue on noncommunicable diseases. Since much is known of the clinical interventions in managing these conditions, this theme issue will be focusing on the political economy and experiences in addressing the commercial drivers of noncommunicable diseases and the impact of international trade on government policy on these diseases.^9^ The commercial drivers are a significant challenge that requires better evidence, multisectoral actions and citizens’ involvement. We welcome all papers that address the industries’ interference in public policies and the gaps between policy and the implementation of population-based best-buy interventions that address modifiable behavioural risk factors.^10^

Understanding the factors that contribute to these bottlenecks will inform effective actions. The multisectoral nature of noncommunicable diseases means that prevention and control go beyond the mandates of health ministries. Policies that ensure physical and social environments conducive to active lifestyles, articles that address policy coherence across sectors as well as the role of civil society organizations and citizens in advocating and increasing public awareness are welcome. Other areas of interests are how universal health coverage could alleviate the burden of noncommunicable diseases and implementation gaps in primary health care, surveillance, monitoring and evaluation for preventing and controlling noncommunicable diseases.

The deadline for submission is 15 July 2018. Manuscripts should be submitted in accordance with the Bulletin’s guidelines for contributors, and the cover letter should mention this call for papers. The theme issue will be launched at Prince Mahidol Award Conference in January 2019.
